# Functional and Genomic insights into probiotic Lactic Acid Bacteria isolated from the Gastrointestinal microbiota of domestic rabbits (O*ryctolagus cuniculus*)

**DOI:** 10.1186/s12866-026-04871-6

**Published:** 2026-04-09

**Authors:** Oluwatosin Olubunmi Oladipo, Abimbola Olumide Adekanmbi, Opeyemi U. Lawal, Valeria R. Parreira, Bolaji Fatai Oyeyemi, Olatunji Abubakar Jimoh, Aderemi Akinyemi, Charles Ayorinde Ologunde, Ayonposi Bukola Olaoye, Olugbenga David Oloruntola, Mitra Soni, Harmanpreet Kaur, Lawrence Goodridge

**Affiliations:** 1 Environmental Microbiology and Biotechnology Laboratory, Department of Microbiology, University of Ibadan, Ibadan, Nigeria; 2 Molecular Biology and Biotechnology Laboratory, Department of Microbiology, University of Ibadan, Ibadan, Nigeria; 3 Department of Science Technology, The Federal Polytechnic Ado-Ekiti, Ado- Ekiti, Ekiti State Nigeria; 4 School of the Environment, University of Windsor, Ontario, Canada; 5 Great Lakes Institutes for Environmental Research, University of Windsor, Ontario, Canada; 6 Canadian Research Institute for Food Safety, University of Guelph, Guelph, ON Canada; 7 Agricultural Technology Department, The Federal Polytechnic Ado Ekiti, Ado Ekiti, Nigeria; 8 Cell Biology and Genetics Unit, Department of Zoology, University of Ilorin, Ilorin, Nigeria; 9 Department of Animal Science, Adekunle Ajasin University, Akungba Akoko, Nigeria

**Keywords:** Probiotics, Rabbit, Lactic acid bacteria, Enterococcus spp., Whole Genome Sequencing

## Abstract

Probiotics play vital roles in maintaining gut health and overall well-being of animals, and have been isolated from the guts of several animals. The caecotrophic and dynamic nature of rabbit gut make them a promising site for probiotic prospecting. This study therefore, investigated the probiotic properties and whole genome sequence profile of Lactic Acid Bacteria (LAB) isolated from the gut microflora of domestic rabbit. Gastrointestinal contents of rabbits were cultured anaerobically on de Mann Rogosa Sharpe (MRS) agar using the pour-plate method. Isolates obtained were initially identified using 16S rRNA sequencing and later using Whole genome sequencing. Probiotic properties of the LAB were determined using conventional in vitro tests such as acid and phenol tolerance, adhesion ability, susceptibility to commonly-used antibiotics and the antimicrobial activity of their cell-free supernatants against selected test microorganisms. Whole genome analysis was carried out on selected isolates to further confirm their probiotic and safety properties.

Three LAB showing probiotic properties were obtained and identified: *Enterococcus lactis* UIADO22, *E. lactis* UIADO37, and *Enterococcus hirae* UIADO30. All isolates were non-haemolytic, DNAse-negative, tolerant to pH 2, and maintaining viability in simulated duodenal juice. They were all sensitive to the tested antibiotics and their cell-free supernatants demonstrated antibacterial activity against selected test bacteria. Whole genome sequencing revealed the absence of vancomycin resistance and virulence genes (*IS16*, *esp*, *hyl*) in the LAB, confirming their safety. Probiotic potential was further supported by the detection of genes encoding bacteriocins such as *Enterolysin_A*,* Subtilosin_A*,* Enterocin_L50a/L50b*,* Lanthionine synthetase*, and *Enterocin_P*. Biosynthetic gene clusters for antimicrobial metabolites, including cyclic-lactone autoinducers, Type III polyketide synthase, terpene precursors, sactipeptides, RIPP-like peptides, and Lanthipeptide Class II, were also detected. Genes associated with stress resistance especially bile, acid and phenol (such as *dltA*, *dltB*, *ssb* and *dltC*), DNA/protein repair (such as *recA*, *uvrA*, *uvrB*,and *recN*), adhesion and anti-pathogenic activity (such *agrB*,* agrD*,* fsrA*,and *fbp3)* were detected in the genomes of the three LAB. Overall, the rabbit-derived *Enterococcus* spp. from this study exhibited both in vitro and genomic characteristics of safe, functional probiotics with strong antimicrobial potential. *Enterococcus hirae* UIADO30 is the most promising isolate because it lacks plasmids and virulence markers thereby reducing the risk of gene transfer—while carrying markers for bile and acid resistance.

**Supplementary Information:**

The online version contains supplementary material available at 10.1186/s12866-026-04871-6.

## Introduction

Probiotic microorganisms are increasingly recognised for their role in promoting host health through mechanisms such as modulation of gut microbiota, enhancement of mucosal barrier integrity, competitive exclusion of pathogens, and immunomodulation [1, 2] Among these, Lactic Acid Bacteria (LAB) remain one of the most extensively studied groups due to their historical use in fermented foods, demonstrated resilience in gastrointestinal conditions, and broad spectrum of beneficial effects [2]. Within LAB, members of the genus *Enterococcus*, have attracted much attention for their dual roles as commensals and probiotics. These isolates possess desirable probiotic traits such as bacteriocin production, epithelial adhesion, and antagonistic activity against certain enteric pathogens [3].

Hindgut fermentation and a complex gut microbiota are crucial for effective digestion and overall health, and the structure and physiology of the rabbit gastrointestinal tract (GIT) are well-suited to promote these processes. The stomach, small intestine, colon, and big caecum make up the rabbit GIT. The caecum is remarkably large in relation to their body size, accounting for around 40% of the GIT volume [4]. The hallmark of the rabbit digestive system is the highly developed caecum, which acts as a fermentation chamber hosting an abundant and heterogeneous microbial community composed of bacteria, protozoa, and fungi that specialize in breaking down fibrous plant materials [5]. The distinct process of caecotrophy (re-ingestion of caecal pellets) permits rabbits to optimise nutrient extraction through further digestion and absorption in the small intestine, underscoring the functional integration of caecal fermentation with overall digestive physiology [6].

On the other hand, the production of enterocins, a potent bacteriocin, by certain *Enterococcus* species makes them outstanding probiotic candidates, particularly when combined with their resilience to survive in the harsh gut conditions [7]. However, there is a need to guide against major concerns such as antibiotic resistance and virulence factors, which could negatively impact their proper function. Traditionally, probiotic candidates have been identified and screened using in vitro phenotypic assays. More recently, Whole Genome Sequencing (WGS) has emerged as a complementary approach, providing genetic evidence that strengthens both in vivo and in vitro findings [7]. Probiotics have been isolated from diverse animal sources and screened using WGS. For example, Lengliz et al. [8] reported probiotic strains from wild and laboratory rabbit faeces, while Fu et al. [9] identified *Enterococcus* species from healthy individuals. Kereszteny et al. [10] isolated probiotic strains from wild boars, while Zaghloul and Halfway [11] recovered *Pediococcus* species from shrimp gut. Similarly, Nyamaiofofe et al. [12] obtained *Enterococcus* and *Lactococcus* strains from Wistar rat faeces, whereas Prakitit et al. [13] described two novel probiotic *Heyndrickia coagulans* strains from stingless bee honey. Whole-genome annotation has revealed genes essential for probiotic functions in microorganisms, especially *Enterococcus* spp. [7, 14]. The combination of phenotype-genome based characterisation is the way forward in assessing the safety of probiotics, as this combination approach would greatly improve the safety and efficacy of potential probiotics [7, 15].

While LAB have been extensively isolated from humans and livestock, the GIT of domestic rabbits (*Oryctolagus cuniculus*) remains underexplored as a reservoir of novel probiotic strains especially in Nigeria. Rabbits possess a unique hindgut fermentation system that supports a diverse and functionally rich microbiome [16], yet few studies have characterised their indigenous LAB for probiotic use. This gap is particularly relevant in the context of functional feed development for companion animals and sustainable agriculture. Moreover, combining in vitro phenotypic assays with WGS offers a robust strategy for identifying and validating safe, functionally potent probiotic strains [14]. Phenotypic screening enables the detection of probiotic properties of the LAB (acid and bile tolerance, antimicrobial activity, and adhesion ability), with the genome-based approach offering a validation of these characters. In this study, we isolated LAB strains from the GIT of domestic rabbits and subjected them to a comprehensive phenotypic and genomic characterisation to evaluate their probiotic potential and assess their safety profiles as promising probiotic candidates.

## Materials and methods

### Ethical approval

Ethical approval for this study was obtained from the Animal Care and Ethics committee of The Federal Polytechnic, Ado-Ekiti with approval number (FPA/EC/20/0062). All animal experiments followed both local and international guidelines and protocols.

### Sample collection, isolation, and preliminary identification of LAB

Samples were obtained from gastrointestinal content of forty apparently healthy, foliage-fed domestic rabbits (*Oryctolagus cuniculus*). The animals were sourced with full and informed consent from the Rabbit flock of the Teaching and Research farm of the Department of Agricultural Technology, The Federal Polytechnic, Ado-Ekiti. Animals were anesthetized with ketamine (20 mg/kg, intramuscular). Adequate anesthetic depth was confirmed by the absence of pedal and corneal reflexes. This was followed by exsanguination under deep anesthesia, ensuring that animals did not regain consciousness. This procedure was conducted in accordance with established Animal welfare and Euthanasia guidelines [17]. The gastrointestinal samples were collected into sterile containers, transported on ice to the Laboratory, and processed within three hours of collection. Samples were plated out on de Man Rogosa and Sharpe (MRS) agar, after serial dilution was carried out on the samples. Incubation was done anaerobically at 35 ± 2 °C for 48 h in an anaerobic jar to which AnaeroGen (Oxoid, UK), an anaerobic condition generator has been incorporated [17]. Colonies showing the colonial morphology typical of LAB were selected from the plates representing each rabbit, subcultured to obtain pure cultures and stored on agar slant kept at 4 °C for short-term storage and glycerol broth kept at -80 °C for long-term storage. Preliminary identification of presumptive LAB isolates was done using conventional methods including morphological, sugar fermentation and biochemical tests, following standard methods [18].

### Screening for probiotic potential of the LAB isolates

#### Haemolytic activity, DNAse test, and resistance to phenol

Haemolytic activity was assessed by streaking the isolates on blood agar plates containing 5% (v/v) whole human blood and incubating at 35 ± 2 °C for 48 h. Haemolysis was interpreted as α, β, or γ (non-haemolytic) based on visual inspection [19]. DNase activity was carried out on DNase agar, while tolerance to phenol (0.4%) was evaluated using the method described by Jena et al. [20], with slight modifications.

#### *Antibiotic susceptibility testing*

The susceptibility of the LAB to six classes of antibiotics was determined using the disc diffusion method according to Kirby-Bauer [21].The antibiotics used were purchased from Oxoid, United Kingdom, and included penicillin (10 µg) ampicillin (10 µg), imipenem(10 µg), gentamicin (10 µg), vancomycin (30 µg), erythromycin (15 µg), and clindamycin (2 µg). Overnight culture of LAB was introduced into 5mL of 0.85% normal saline and adjusted to 0.5 McFarland standard. This was introduced by swabbing to already-prepared MRS agar plates. Discs of the antibiotics were placed on the inoculated plates using a sterile pair of forceps and incubated anaerobically at 35 ± 2 °C for 24 h. Zones of inhibition around the discs were measured and recorded [22].

### Antimicrobial activity of the cell-free supernatants of the LAB

Antimicrobial activity of the cell-free supernatants of the LAB was evaluated using the agar well diffusion method [23]. Test bacteria used included *Escherichia coli* ATCC 25922, *Staphylococcus aureus* ATCC 29213, *Bacillus subtilis* ATCC 6633 and *Klebsiella pneumoniae* ATCC 70303, while the fungi used were *Aspergillus niger* and *Penicillium sp..* Overnight cultures of the test bacterial isolates were inoculated into sterile normal saline and standardized to 0.5 McFarland turbidity. The standardized suspension of the isolates was swabbed onto Mueller-Hinton agar (Oxoid, UK) plates using sterile swab sticks, and 50 µL of sterile cell-free supernatants was added to already-perforated wells. Zones of inhibition were measured after 24-hour incubation at 35 ± 2 °C. For the fungi, Potato Dextrose Agar (Oxoid, UK)) plates were inoculated with pure fungal cultures to form a lawn, and agar wells were made using a sterile cork borer. The wells were filled with the cell-free supernatants of the LAB, and plates were incubated at room temperature for 48-72 hours. Zones of inhibition were measured and recorded. 

### Acidic pH tolerance

The ability of the LAB to tolerate acidic pH was tested using the modified method of Sahadeva et al. [24]. The test was carried out using Phosphate Buffer Saline (PBS) solution, with pH adjusted to 2 using 1 M HCl. Aliquot (1mL) of the LAB containing approximately 10^6^ cells mL^− 1^ was inoculated into 9mL of PBS, and incubated for 0, 60, and 120 min. At the end of the incubation period, the set-up was plated on MRS agar, incubated anaerobically for 48 h, and the bacterial load in the set-up and control plates recorded.

### Survival in simulated duodenum juice (bile salt tolerance)

The bile salt tolerance was done using the methods described by Faye et al. [25]. and Anandharaj et al. [26]. Aliquot (1mL) of freshly prepared overnight culture of the LAB was inoculated in artificial duodenum juice containing the following (NaCl-1.28 g KCl-0.239 g, NaHCO_3_ 6.4 g, Bile salt-0.3%, pepsin-0.1 g) in 1000mL of distilled water and maintained at a pH of 2.5. Incubation was carried out at 35 ± 2 °C for 0, 2, and 4 h, with phosphate buffer serving as the control. Viable cell count was determined and recorded as CFU/ml.

### Cell surface hydrophobicity

The hydrophobicity of the cell surface of the LAB isolates was assessed in vitro by measuring the cell adhesion to hydrocarbons using n-hexane and xylene, according to the protocol outlined by Kalyaraung et al. [27] and Rokana et al. [28]. LAB isolates were cultured overnight, harvested by centrifugation, and washed three times with phosphate buffer (pH 6.5). The bacterial pellet was resuspended in the buffer to an optical density (OD) of approximately 0.4 at 450 nm. Subsequently, 0.6 mL each of n-hexane and xylene was separately added to 3 mL of the bacterial suspension. The mixture was vortexed intermittently and incubated at 37 °C for 15 min, followed by a 25-minute rest at room temperature to allow phase separation. The aqueous phase was carefully collected, and its absorbance was measured at 450 nm. The percentage of cell surface hydrophobicity was calculated using the formula: Hydrophobicity (%) = [(OD_initial – OD_final) / OD_initial] × 100, where OD_initial is the absorbance before solvent addition and OD_final is the absorbance after phase separation. All measurements were performed in triplicate to ensure reproducibility. The adhesion percentage for each compound was computed and recorded.

### Molecular identification of isolates using 16S rRNA gene sequencing

The three isolates of the thirty-seven obtained that passed the in vitro tests for probiotic activities were selected for 16S rRNA Sequencing and Whole Genome Sequencing. The isolates were cultured in MRS broth and incubated overnight. Genomic DNA was extracted using the Zymo DNA Miniprep Kit (Zymo Research, USA). The 16S rRNA gene was amplified using primers 27 F (AGAGTTTGATCMTGGCTCAG) and 519 R (GWATTACCGCGGCKGCTG) following PCR conditions described by Chennappa et al. [29]. Amplicons were resolved on 1% agarose gel, purified, and sequenced. Sequence identities were confirmed using NCBI BLAST, and phylogenetic relationships were inferred using MEGA 5.1 software with the Maximum Likelihood method. The resulting sequences were deposited in the GenBank for the assignment of Accession Numbers.

### Whole genome sequencing, assembly, and annotation

Subculturing of Isolates and Characterisation: Purified colonies were selectively collected and subjected to biochemical identification using the VITEK 2© system (BioMérieux, Canada) following the manufacturer’s instructions. Species identification was considered valid only when the confidence level was ≥ 85%, and only colonies meeting this criterion were retained for further analysis.

### DNA extraction and genomic library preparation

Genomic DNA was extracted using the DNeasy Blood and Tissue Kit (Qiagen) according to the manufacturer’s protocol for Gram-positive bacteria, which includes a lysozyme pretreatment step to enhance cell lysis. DNA quantity and quality were assessed using a QIAexpert spectrophotometer (Qiagen) and Qubit fluorometer (Invitrogen), and high-quality DNA samples were subjected to WGS. Genomic DNA libraries were prepared using the Illumina DNA prep tagmentation kit (#20018704) and IDT for Illumina DNA/RNA UD indexes (#20027213) following the manufacturer’s instructions.

### Sequencing and bioinformatics analysis

Paired-end (2 × 150 bp) sequencing was performed using the high-output flow cell on the Illumina NextSeq 1000 instrument. Raw reads were quality-filtered, trimmed, and assembled as previously described Lawal et al. [30]. Genome annotation was performed using Prokka (v1.14.5) [31] and the NCBI Prokaryotic Genome Annotation Pipeline [32]. Mobile genetic elements and plasmid-associated sequences were identified using Mobile Element Finder [33] and Plasmid Finder [34] respectively. PHASTEST was used to predict prophage regions [35] while regions containing clustered regularly interspaced short palindromic repeats (CRISPR) were identified using the CRISPR Finder server [36]. Proskee.ca was used for in-depth characterisation and visualisation of the bacterial genome, showing the circular chromosome and genome stability [36].

### Genome-based safety assessment

To assess probiotic safety at the genomic level, all three isolates were screened for antimicrobial resistance (AMR) and virulence genes. BLASTP searches were conducted against the Virulence Factors Database (VFDB) and Comprehensive Antibiotic Resistance Database (CARD), as previously described by Alcock et al. [37]. Genes linked to haemolysins, aggregation substances, cytolysins, and transferable resistance elements were critically examined. Genomic context and plasmid association of any detected AMR genes were also evaluated to assess mobility and potential risk [38].

### Assessment of probiotic properties

#### Analysis of antimicrobial compounds in the genomes of the LAB

Bacteriocin gene clusters and antimicrobial compounds were identified and annotated using BAGEL5, a web-based platform specifically developed for detecting ribosomally synthesized and post-translationally modified peptides (RiPPs), including bacteriocins [39]. The presence of antimicrobial biosynthetic gene clusters was analyzed using antiSMASH version 8.0, a tool designed for the identification and annotation of secondary metabolite biosynthesis pathways [40].

#### Identification of genes associated with probiotic activities

Probiotic-associated genes were identified *in-silico* by screening for functional markers associated with adhesion, stress resistance, antioxidative defense, immunomodulation, and pathogen antagonism. Protein sequences were compared via BLASTP to curated probiotic gene databases, and functional roles, namely adhesion ability, active stressor removal, DNA and protein protection and repair, stress resistance, and anti-pathogenic effects were assigned as described by Zheng et al. [38]. 

## Results

### Frequency of LAB obtained from the gastrointestinal tract of rabbits

A total of thirty-seven presumptive LAB were obtained from the gastrointestinal content of forty apparently healthy domestic rabbits. The morphological and biochemical characterisation of the isolates confirmed them as presumptive LAB. They were all gram positive cocci, catalase, oxidase and indole negative. They were able to reduce nitrate and unable to utilise citrate.

#### Molecular identification of the LAB isolates using 16S rRNA and WGS

The identities of the three selected LAB isolates using 16S rRNA gene sequencing and WGS, though still the same generically, do not the same species annotation. The 16S rRNA sequencing identified the isolates as *Enterococcus faecium* UIADO22, *Enterococcus hirae* UIADO30, and *Enterococcus durans* UIADO37, while WGS reassigned UIADO22 and UIADO37 as *Enterococcus lactis* while confirming UIADO30 as *Enterococcus hirae*. The WGS-based identities (*E. lactis* UIADO22, *E. hirae* UIADO30, and *E. lactis* UIADO37) are used hereafter as shown in Table 1.

**Table 1:** Identity of the isolates using 16S rRNA sequencing and whole genome sequencing

Isolate Code	16S rRNA	Whole Genome Sequencing
UIADO22	*Enterococcus faecium*	*Enterococcus lactis*
UIADO30	*Enterococcus hirae*	*Enterococcus hirae*
UIADO37	*Enterococcus durans*	*Enterococcus lactis*

### Genome features and annotation of the three selected LAB

**Fig. 1:**
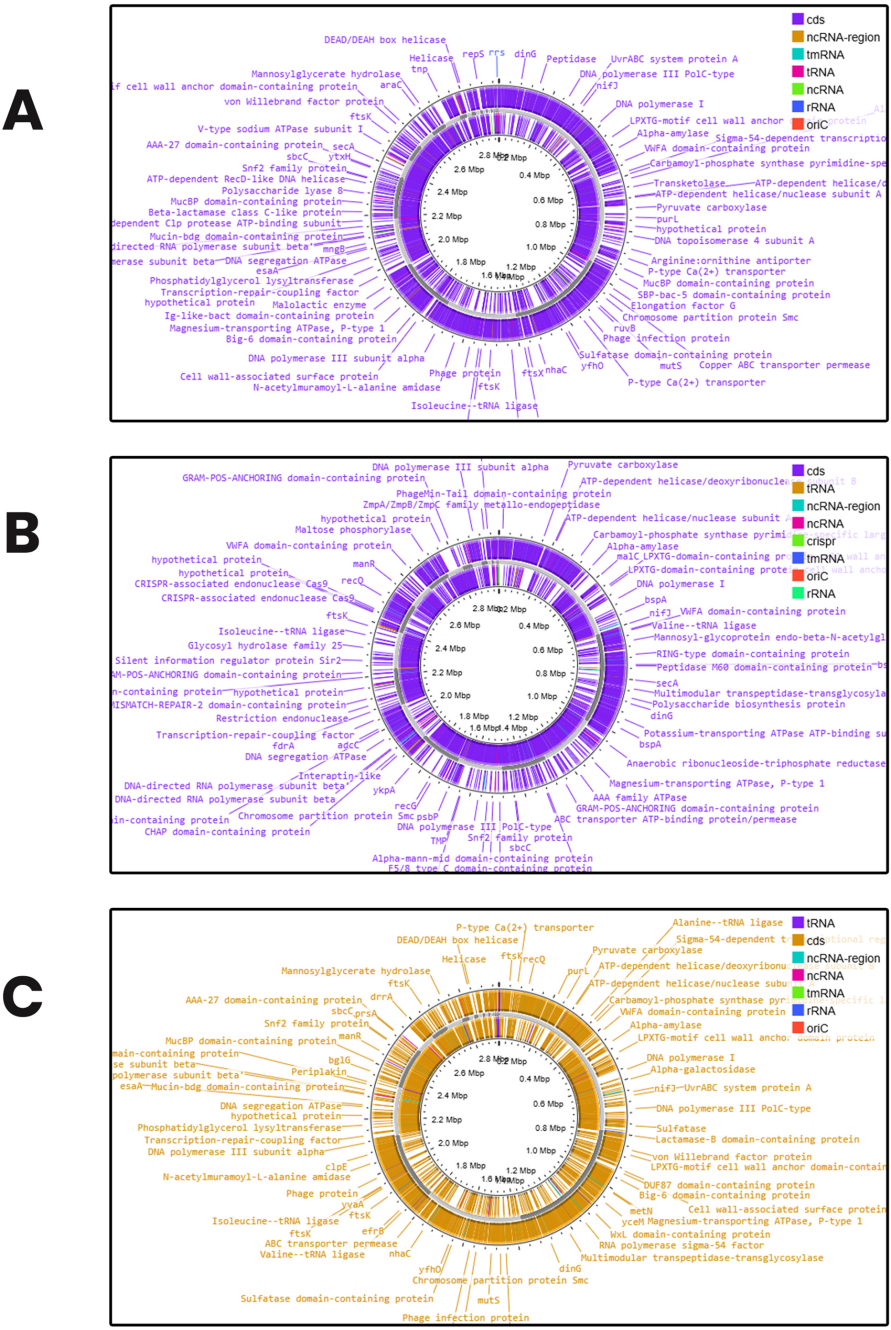
Circular chromosome of (**a**) *Enterococcus lactis* UIADO22 (**b**) *Enterococcus hirae* UIADO30 (**c**) *Enterococcus lactis* UIADO37

The circular chromosomes of the genomes are shown in Fig. 1. The Genome size for *Enterococcus lactis* UIADO22 was 2,942,513 bp, 2,990,747 bp for *Enterococcus hirae* UIADO30, and 2,942,524 bp for *Enterococcus lactis* UIADO37. The number of contigs was 37 in *Enterococcus lactis* UIADO22, 29 in *Enterococcus hirae* UIADO30, and 34 in *Enterococcus lactis* UIADO37. The Guanine and Cytosine (GC) content was 38.05% for *Enterococcus lactis* UIADO22 and *Enterococcus lactis* UIADO37, while it was 36.69% for *Enterococcus hirae* UIADO30. No CRISPR system was found in both *Enterococcus lactis* UIADO22 and *Enterococcus lactis* UIADO37, but two were found in *Enterococcus hirae* UIADO30. The number of coding DNA sequences is 2780, 2707, and 2779 for *Enterococcus lactis* UIADO22, *Enterococcus hirae* UIADO30, and *Enterococcus lactis* UIADO37, respectively. There were no GAP regions in the genomes of the three organisms. Two origins of chromosomal replication (*oriC*) were found in *Enterococcus lactis* UIADO22 and *Enterococcus lactis* UIADO37, while only one was found in *Enterococcus hirae* UIADO30. No origin of transfer (*oriT*) was found in the three genomes.

### LAB isolates from rabbit gut exhibit antimicrobial and probiotic potential

#### Antibiotic susceptibility profiling and haemolytic activity

Table S1 shows the antibiotic susceptibility profile of the LAB isolates to a panel of selected antibiotics. The three isolates did not show any observable level of resistance to the tested antibiotics, as they all showed different levels of sensitivity. All the antibiotics used were effective in inhibiting the growth of the three LAB isolates. Table S2 shows the haemolytic activity of the three selected LAB isolates on blood agar and their response to DNAse medium. They were all non-haemolytic and did not show hydrolytic activity on DNAse medium.

#### Antibiotic resistance and virulence genes found in the genomes of the three LAB isolates

The antibiotic resistance and virulence genes detected in the genomes of the three LAB isolates are shown in Table 2. Aminoglycoside resistance gene (*aac(6’)-Ii*) and *msrC*, which encodes macrolide resistance were found in the genomes of *Enterococcus lactis* UIADO22 and *Enterococcus lactis* UIADO37, while *aac(6’)-Iid and tet(O)* encoding aminoglycoside and tetracycline resistance respectively, were found in the genome of *Enterococcus hirae* UIADO30. These antibiotic resistance genes are intrinsic to *Enterococcus* species and are not located on any mobile genetic element. Two virulence genes, *acm* (encoding a collagen-binding adhesin, which is important for colonization and biofilm formation) and *sgrA* (encoding a surface protein, which is an adhesin, playing a role in biofilm formation) were found in the genomes of *Enterococcus lactis* UIADO22 and *Enterococcus lactis* UIADO37. The two genes were not detected in the genome of *Enterococcus hirae* UIADO30.

**Fig. 2:**
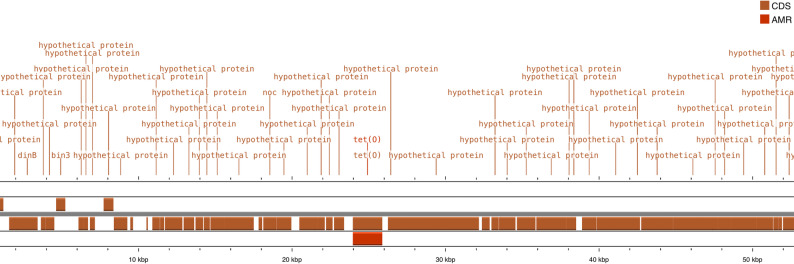
The upstream and downstream genes surrounding *tetO* gene

Figure 2 shows the upstream and downstream genes around the location of the *tetO* gene. The Figure indicates the absence of mobile genetic elements, nearby insertion sequences, or mobilizable plasmids capable of facilitating gene transfer, thereby alleviating concerns about safety risks when used as probiotics.

**Table 2:** Antibiotic resistance and virulence factors carried by the probiotic LAB

Antibiotic resistance gene	Gene function	*Enterococcus lactis* UIADO22	*Enterococcus hirae* UIADO30	*Enterococcus lactis* UIADO37	Percentage identity
*aac(6’)-ii*	aminoglycoside resistance	+	-	+	100
*aac(6’)-iid*	aminoglycoside resistance	-	+	-	100
*msrC*	msr-type ABC-F protein for resistance to macrolide and streptogramin	+	-	+	100
*tet(O)*	tetracycline operator gene	-	+	-	100

Virulence factors	Gene function (Location)	*Enterococcus lactis* UIADO22	*Enterococcus hirae* UIADO30	*Enterococcus lactis* UIADO37	Percentage identity
*Acm*	adhesin of collagen (Chromosome)	+	-	+	73.92
*sgrA*	serine-glycine repeat protein A”/ cell wall anchored protein (Chromosome)	+	-	+	71.28

KEY: +: Present; -:Absent

### Plasmid and other mobile elements

Two plasmids, *rep1_1_repE*(pAMbeta), and *repUS15*_2_*repA*(pNB2354p1) were found in the genomes of *Enterococcus lactis* UIADO22 and *Enterococcus lactis* UIADO37 with over 95% identity, while none was found in the genome of *Enterococcus hirae* UIADO30. The position of the plasmids and their similarity to known plasmids are shown in Supplementary Table S3. The number of prophage regions in the genomes of *Enterococcus lactis* UIADO22, and *Enterococcus lactis* UIADO37 is one, and they are intact with 37.7 kb size, while there are four prophage regions in *Enterococcus hirae* UIADO30, with three intact regions and one incomplete region with the following sizes 17.0, 59.5, 15, and 13.6 kb, respectively as shown in Supplementary Table S4.

#### Antimicrobial activity of the cell-free supernatant of the LAB isolates

Table 3 shows the activity of the cell-free supernatant of the three probiotic LAB against selected test bacteria and fungi. There were zones of inhibition against all the bacteria , with values ranging from 9.1 to 15 mm for all the isolates tested. The highest zone of inhibition was observed against *Bacillus subtilis* (15 mm), with the cell-free supernatant of *Enterococcus lactis* UIADO37. However, none of the fungi used was inhibited by the cell-free supernatants of the three LAB.

**Table 3:** Inhibition of the growth of selected microorganisms by the cell-free supernatants of the probiotic LAB (mm)

Isolate	EC	SA	KP	BS	AN	PS
*Enterococcus lactis* UIADO22	11	10.5	9.2	13	NA	NA
*Enterococcus hirae* UIADO30	12	12	8.5	12	NA	NA
*Enterococcus lactis* UIADO37	12.5	12	9.1	15	NA	NA

KEY: *EC* *Escherichia coli*, *SA* *Staphylococcus aureus*, *KP* *Klebsiella pneumoniae, BS* *Bacillus subtilis*, *AN* *Aspergillus niger*, *PS* *Penicillium* sp*.*, *NA *No Activity

### Genes encoding bacteriocin production and antimicrobial compounds in the three selected LAB

**Fig. 3:**
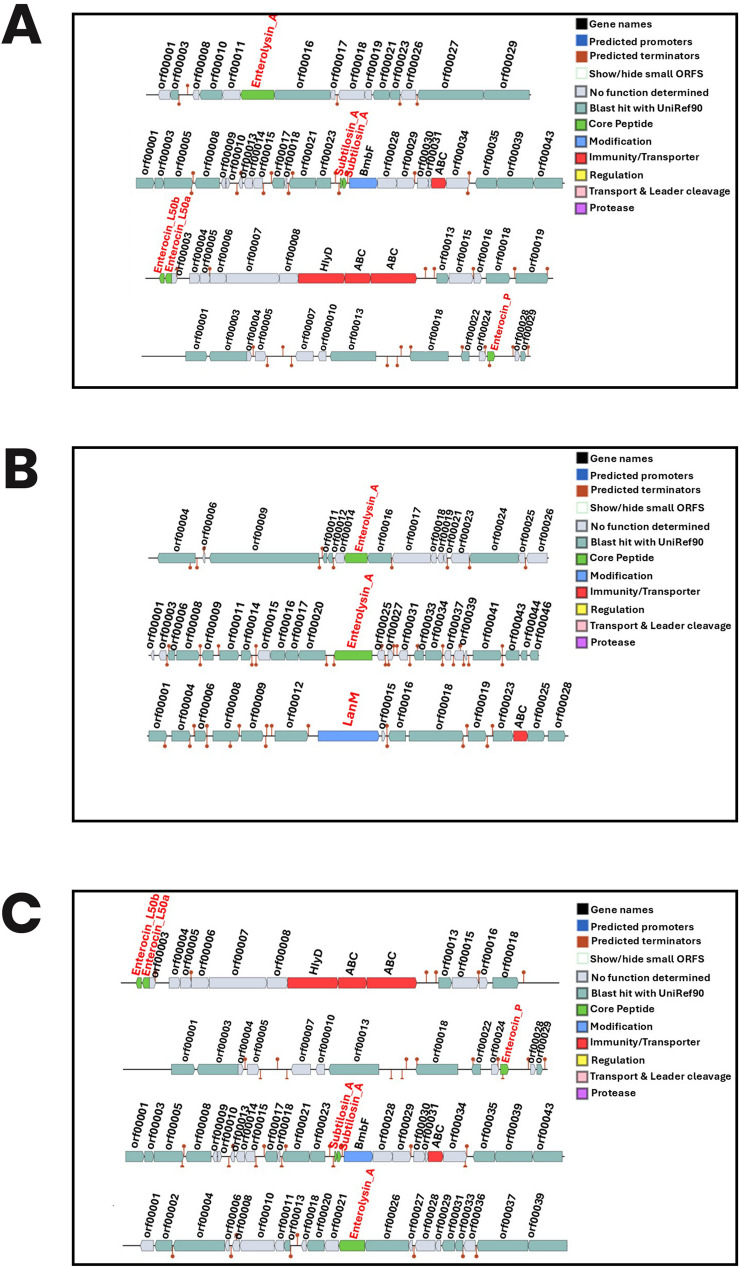
Bacteriocins produced by (**a**) *Enterococcus lactis* UIADO22 (**b**) *Enterococcus hirae* UIADO30 and (**c**) *Enterococcus lactis* UIADO37

**Fig. 4:**
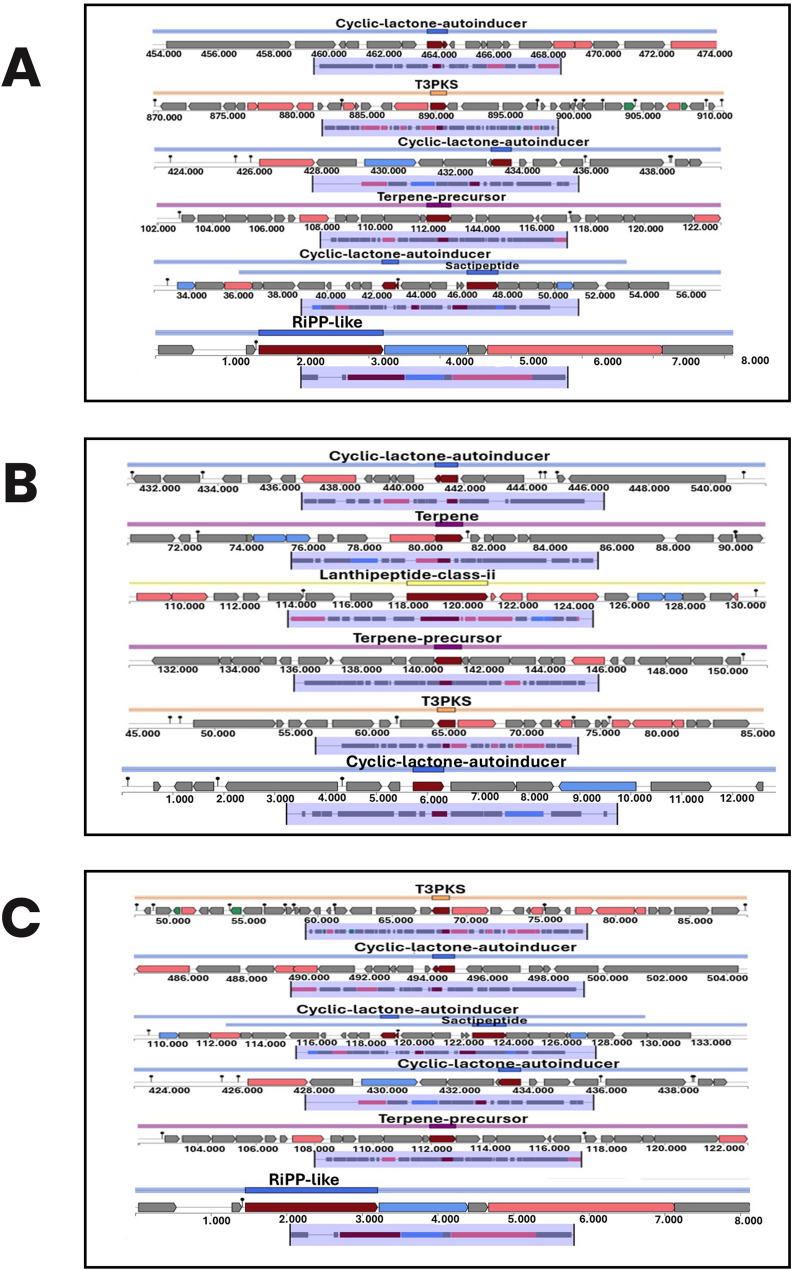
Biosynthetic gene clusters encoding antimicrobial compounds found in the genomes of the three organisms (**a**) *Enterococcus lactis* UIADO22 (**b**) *Enterococcus hirae* UIADO30 (**c**) *Enterococcus lactis* UIADO37

Genomic analysis predicted the presence of multiple genes associated with the production of antimicrobial compounds and bacteriocins in the three isolates. Figure 3 shows that bacteriocin-encoding genes; *Enterolysin_A*,* Subtilosin_A*,* Enterocin _L50b*,* Enterocin _L50a*, and *Enterocin_p* were found in *Enterococcus lactis* UIADO22 and *Enterococcu*s lactis UIADO37, while *62.3 Enterolysin_A*,* 63.3 Enterolysin_A*, and *Lanthionine synthetase* (*Lan M*) were found in *Enterococcus hirae* UIADO30. Figure 4 shows the biosynthetic gene clusters (BGCs) in the isolates. Six clusters were each found in *Enterococcus lactis* UIADO22 and *Enterococcus lactis* UIADO37, and five clusters in *Enterococcus hirae* UIADO30. They are predicted to be associated with the production of antimicrobial compounds.

#### LAB isolates from rabbit gut exhibited probiotic properties vis-à-vis the carriage of probiotic-encoding genes

**Fig. 5:**
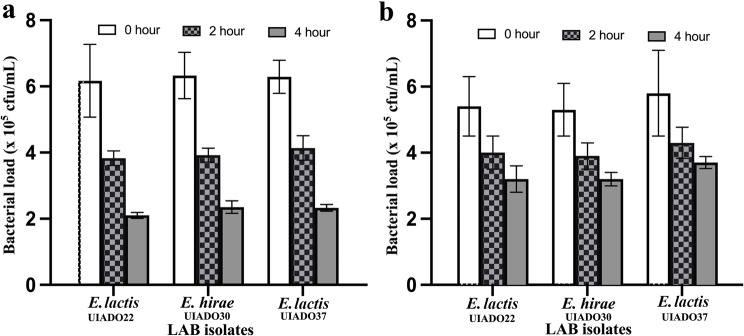
Viable cells of the LAB isolates (**a**) at pH 2 and (**b**) in simulated duodenum juice

A very important characteristic for any isolate with probiotic potential is its tolerance to acidic pH. The isolates from this study were able to tolerate acidic pH of 2, albeit with the viable cell number reducing with increasing period of incubation. There was a marked reduction in the number of viable cells of the LAB isolates compared to the control, as the incubation time increased from 0 to 4 h (Fig. 5a).

There was a significant reduction in the bacterial load in the simulated duodenum juice for the first two hours of incubation. The highest viable number observed was for *Enterococcus lactis* UIADO37 with 4.3 × 10^5^ cfu/mL from the initial starting load of 5.8 × 10^5^ cfu/ml. By the end of incubation at the fourth hour, both *Enterococcus lactis* UIADO22 and *Enterococcus hirae* UIADO30 had the same number of viable cells (3.7 × 10^5^ cfu/mL). For the three isolates, the percentage reduction in the viable cells was *Enterococcus lactis* UIADO22 (40.74%), *Enterococcus hirae* UIADO30 (39.62%) and *Enterococcus lactis* UIADO37 (36.20%), making *Enterococcus lactis* UIADO37 the most tolerant of the organisms to the simulated duodenum juice (Fig. 5b). The three isolates from this study were able to grow and tolerate phenol at 0.4% concentration. 

#### LAB isolates from rabbit gut showed optimum cell surface hydrophobicity

All the three isolates showed optimum hydrophobicity to cell surfaces when exposed to n-hexane and xylene, suggesting that they have probiotic potential. *Enterococcus lactis* UIADO37 had the maximum adhesion with 51.5%, followed by *Enterococcus hirae* UIADO30 (50%) and *Enterococcus lactis* UIADO22 (41.8%) in that order (Table 4).

**Table 4:** Cell surface hydrophobicity of the LAB isolates

Isolate name	*n*-Hexane	Xylene	% Adhesion
*Enterococcus lactis *UIADO22	40.9	42.6	41.8
*Enterococcus hirae *UIADO30	48.3	51.7	50.0
*Enterococcus lactis* UIADO37	52.4	50.5	51.5

### Probiotic-related genes in the genomes of the LAB

Analysis of the three LAB genomes identified a broad repertoire of genes linked to probiotic functionality (Table 5). Notably, multiple stress resistance genes—including *dltA*, *dltB*, *dltC*, *dltD*, *dnaK*, *spxA*, *rex*, *perR*, and *gls*—were detected, which have been reported to enhance tolerance to harsh gastrointestinal conditions such as bile salts, low pH, and phenolic compounds. Genes associated with DNA and protein repair (*recA*, *uvrA*, *uvrB*, *mutS*, and *mutL*) were also present, suggesting that the isolates possess mechanisms to preserve genomic stability under stress. Additionally, the identification of molecular chaperone genes (*groEL*, *HSPD1*, *HSP60*, *CPN60*, and *dnaK*) supports their ability to protect and refold damaged proteins, thereby promoting survival in bile- and phenol-rich environments. The detection of anti-pathogenic regulatory genes (*agrB*, *agrD*, and *fsrA*) indicates potential antagonistic activity against competing or pathogenic microorganisms. Moreover, the presence of the adhesion-related gene *fbp3* suggests that these LAB strains are capable of adhering to host epithelial surfaces, a critical feature for effective colonization and probiotic performance.

**Table 5:** Probiotic-related genes found in the genomes of the three probiotic LAB

Gene	*Enterococcus lactis* UIADO22	*Enterococcus hirae* UIADO30	*Enterococcus lactis* UIADO37	Response
	Locus tag	Locus tag	Locus tag	
*Stress resistance genes*				
* dltA*	N_Entero_22p_14500	N_Entero_30p_09880	N_Entero_37p_18690	D-alanine–poly(phosphoribitol) ligase subunit
* dltB*	N_Entero_22p_14510	N_Entero_30p_09870	N_Entero_37p_18700	membrane protein involved in D-alanine export
* dltC*	N_Entero_22p_14520	N_Entero_30p_09860	N_Entero_37p_18710	D-alanine–poly(phosphoribitol) ligase subunit 2
* dltD*	N_Entero_22p_14530	N_Entero_30p_09850	N_Entero_37p_18720	D-alanine transfer protein
* dnaK*	N_Entero_22p_02870	N_Entero_30p_14170	N_Entero_37p_06530	HSPA9; molecular chaperone
* spxA*	N_Entero_22p_08660	N_Entero_30p_13580	N_Entero_37p_05210	regulatory protein spx
* Rex*	N_Entero_22p_19020	N_Entero_30p_05720	N_Entero_37p_10670	redox-sensing transcriptional repressor
* perR*	N_Entero_22p_06180	N_Entero_30p_02500	N_Entero_37p_03220	Fur family transcriptional regulator, peroxide stress response regulator
* glsA*	N_Entero_22p_04620	N_Entero_30p_10550	N_Entero_37p_04780	GLS; glutaminase
DNA and Protein Protection repair				
* recA*	N_Entero_22p_19880	N_Entero_30p_19090	N_Entero_37p_21530	recombination protein RecA
* uvrA*	N_Entero_22p_02940	N_Entero_30p_14120	N_Entero_37p_06460	excinuclease ABC
* uvrB*	N_Entero_22p_02930	N_Entero_30p_14130	N_Entero_37p_06470	uvrB; excinuclease ABC
* mutS*	N_Entero_22p_10740	N_Entero_30p_16360	N_Entero_37p_14920	DNA mismatch repair protein
* mutL*	N_Entero_22p_10750	N_Entero_30p_16350	N_Entero_37p_14930	DNA mismatch repair protein
* clpP*,* CLPX*	N_Entero_22p_14060	N_Entero_30p_05860	N_Entero_37p_18240	ATP-dependent Clp protease, protease subunit
* clpX*,* CLPX*	N_Entero_22p_03150	N_Entero_30p_05430	N_Entero_37p_06250	ATP-dependent Clp protease ATP-binding subunit
* groEL*, *HSPD1*,* HSP60*,* CPN60*	N_Entero_22p_21910	N_Entero_30p_27540	N_Entero_37p_23560	chaperonin GroEL [EC:5.6.1.7]
* dnaK*	N_Entero_22p_02870	N_Entero_30p_14170	N_Entero_37p_06530	HSPA9; molecular chaperone
* recN*	N_Entero_22p_14850	N_Entero_30p_22440	N_Entero_37p_19040	DNA repair protein RecN (Recombination protein N)
* Ssb*	N_Entero_22p_27210	N_Entero_30p_17790	N_Entero_37p_24820	single-strand DNA-binding protein
Modulation of gene expression				
* agrB*	N_Entero_22p_17300	N_Entero_30p_27970	N_Entero_37p_17790	accessory gene regulator B
* agrD*	N_Entero_22p_17310	--	--	AgrD protein
* fsrA*	N_Entero_22p_17810	N_Entero_30p_27990	N_Entero_37p_09460	two-component system, LytTR family, response regulator AgrA
Adhesion Ability				
* fbp3*	N_Entero_22p_19390	--	N_Entero_37p_11040	fructose-1,6-bisphosphatase III

## Discussion

The demand for alternatives to antibiotics and other antimicrobial compounds in livestock production, particularly in poultry farming, for managing infections, is increasing [41]. The advocacy for probiotics in the management of farm animals has been favoured in so many quarters. This study was carried out to determine the probiotic properties of Lactic Acid Bacteria (LAB) isolated from the gastrointestinal tract of domestic rabbits (*Oryctolagus cuniculus*). Few studies have reported on the isolation of probiotic LAB strains from rabbits, making this study unique and timely [42, 43].

In this study, three LAB with probiotic properties were obtained from the intestinal content of domestic rabbits, which can be linked to their unique characteristics. The hindgut fermentation in the ceacum makes it a good habitat for the growth of LAB [5]. The process of caecotrophy also affects the physiology of rabbits, which makes it a good source for Probiotic LAB [6]. The isolates all belonged to the *Enterococcus* genus and were identified as *Enterococcus lactis* UIADO22, *Enterococcus hirae* UIADO30, and *Enterococcus lactis* UIADO37. *Enterococcus lactis* UIADO22 and *Enterococcus lactis* UIADO37 both exhibited very close sequence similarity (87.75% and 87.65%, respectively), indicating that the two genomes are functionally and evolutionarily identical in the regions compared, despite being isolated from different animals [44]. The isolation of *Enterococcus* spp. with probiotic properties in this study aligns with other studies that have isolated *Enterococcus* spp. with probiotic properties from various sources [7, 45, 46]. All three isolates were negative for haemolytic activity, which is an important factor used in the selection for probiotics as reported by Chaichana et al. [47]. The three *Enterococcus* spp. also showed resistance to 0.4% phenol when it was incorporated into the growth medium.

The whole genome sequence analysis predicted two virulence genes in *E. lactis* UIADO22 and *E. lactis* UIADO37, while no virulence gene was predicted in *Enterococcus hirae* UIADO30. This is slightly different from the works of Fu et al. [48], where they reported eight virulence genes in the genome of *Enterococcus lactis* JDM1, while Chaichana et al. [47]. predicted several virulence genes in the genome of *Enterococcus lactis* RB10. The virulence genes in these two genomes (*acm* and *sgrA*) are mostly involved in bacterial adhesion more than virulence, as they assist the bacteria to bind tightly to the host’s extracellular matrix component, thereby colonising the intestinal mucosa [47, 49]. *E. lactis* UIADO22, *E. hirae* UIADO30, and *E. lactis* UIADO37 did not possess Insertion Sequence 16 (*IS16*), enterococcal surface protein (*esp)*, and hyaluronidase (*hyl)* virulence genes. Thus, they can be categorised as safe and can be used as a potential feed additive according to the European Food Safety Authority (EFSA). Despite the rise in Enterococcal infections globally, no infections have been reported to be associated with *E. lactis* [50].

The antibiotic susceptibility of the three LAB in this study showed that the isolates were all sensitive to the tested antibiotics (penicillin, ampicillin, imipenem, gentamicin, vancomycin, erythromycin, and clindamycin). However, the whole genome sequence analysis identified two genes associated with antibiotic resistance in the genomes of the three LAB with 100% similarity. Aminoglycoside (*aac(6’)-Ii)* and macrolide (*msrC*) resistance genes were detected in *E. lactis* UIADO22 and *E. lactis* UIADO37, while the genome of *E. hirae* UIADO30 carried *aac(6’)-Iid* and tetracycline resistance (*tetO)* genes. The enterococcal intrinsic chromosomal gene *msr(c)*, and (*aac(6’)-Ii)*, which are involved in mediating natural resistance to macrolides and aminoglycosides and detected in *E. lactis* UIADO22 and *E. lactis* UIADO37, are chromosomal, and the gene environment analysis showed that they are not transferable thereby raising no obvious public health concern to their use as probiotics. This is in line with the works of Chaichana et al. [47], Fu et al. [48], and Ahmed et al. [50], who discovered *msr(c)*, and (*aac(6’)-Ii) aac(6’)-Iid)* genes in *Enterococcus* species used as probiotics in their studies.

The detection of *tetO* raised a bit of concern because it confers resistance to tetracycline, but the gene environment analysis of the upstream and downstream showed that there were no mobile genetic elements that could facilitate the mobilisation of this gene. Since mobile genetic elements, such as conjugative plasmids, integrases, transposons, and excision sites for phage, were not present in the ten upstream and downstream genes, the *tetO* gene could be termed cryptic and non-transferable. The presence of only two antibiotic resistance genes in the genomes of the three LAB suggests that they do not carry a high burden of resistance determinants. Moreover, the genetic neighbourhood around the detected resistance genes showed no evidence of nearby insertion sequences, integrative and conjugative elements, or small mobilizable plasmids. This is in line with the studies of Miller et al. [51], and Ahmed et al. [50], who reported the stability of the genes (*aac (6′)-Ii* and *msrC*) on the chromosome of the genomes. This combined with the susceptibility of the isolates to clinical antibiotics, suggests a low risk of horizontal gene transfer and aligns with EFSA guidelines for probiotic safety assessment. In addition, vancomycin resistance genes (*vanA*, *vanB*, *vanC*) in *Enterococcus* used as probiotics are a major safety concern, especially when these strains are intended for animal consumption [7], but they were not detected in the genome of the three probiotic LAB in this study and is in line with earlier reports [52, 53].

Mobile genetic elements such as plasmids, prophages play a major role in horizontal gene transfer in bacteria [54]. Two plasmids were found in the genomes of *Enterococcus lactis* UIADO22 and *Enterococcus lactis* UIADO37. These plasmids show high similarity to known LAB plasmids. A *repUS15* sharing 99.71% identity with the *repA*(pNB2354p1) of *Enterococcus faecium* with accession number (CP004064) and rep1 sharing 97.04% identity with *repE*(pAMbeta) of *Enterococcus faecalis* with accession number (AF007787). These two plasmids are similar to the ones found in the genome of *Enterococcus lactis* RB10 [47], as well as the genomes of other *Enterococci*, as revealed by Daza Prieto et al. [53]. The two plasmids found in the genomes of *Enterococcus lactis* UIADO22 and *Enterococcus lactis* UIADO37 are non-conjugative as they do not carry any antibiotic resistance or virulence-related genes. Also, no Origin of Transfer (*oriT*) was present in the two plasmids, confirming that they are non-mobilizable and cannot aid the transfer of potentially harmful genes through horizontal gene transfer. The genomes of *Enterococcus lactis* UIADO22 and *Enterococcus lactis* UIADO37 have one complete prophage region, while the *Enterococcus hirae* UIADO30 has three complete prophage regions and one incomplete region. These prophages did not harbour any virulence or antimicrobial resistance genes, confirming their stability.

*Enterolysin_A*,* Subtilosin_A*,* Enterocin _L50b*,* Enterocin_L50a*,* and Enterocin_p* which are bacteriocin-encoding genes were found in the genomes of *Enterococcus lactis* UIADO22 and *Enterococcus lactis* UIADO37, while 62.3 *Enterolysin_A*, and Lanthionine synthases were found in the genome of *Enterococcus hirae* UIADO30. This is an indication of the antimicrobial properties of the three probiotic LAB. Several studies have reported on bacteriocins produced by *Enterococcus* species showing activity against Gram positive bacteria isolated from diverse environments [7, 48, 55]. *Enterolysin_A*, has a broad spectrum of antimicrobial activities, as it binds to the bacterial cell wall, hydrolysing the peptidoglycan layer, and leading to cell lysis [56]. *Subtilosin_A* is mostly found in *Bacillus subtilis*, its presence in the genomes of *Enterococcus lactis* UIADO22 and *Enterococcus lactis* UIADO37 suggests a broad-spectrum activity and ecological migration among species. *Subtilosin_A* belongs to the lantibiotics class of bacteriocins and has anti-biofilm activity [57]. Various bacteria, including species of the genera *Streptococcus*, *Staphylococcus*, and *Bacillus*, produce lanthionine synthetase.

On the other hand, *Enterocin P* is mainly active against Gram positive bacteria and targets the cytoplasmic membrane of bacteria, leading to cell lysis. Additionally, bacteriocin production has been important for selecting probiotic strains, since they can inhibit the invasion of potential pathogens, and in the process positively modulating the host microbiota. Lanthipeptide_class_II and *Enterolysin_A* were found in a study carried out by Li et al. [43]. The genome analysis of *E. hirae* R44 predicted the presence of antimicrobial compounds like *Enterolysin_A*, class II lanthipeptide, and terpenes, which underlay its antibacterial attributes. These findings suggest that *E. lactis* UIADO22, *E. hirae* UIADO30 *and E. lactis* UIADO37 may have probiotic properties that could benefit the gastrointestinal environment.

The LAB in this present study showed antibacterial activity in vitro against selected test organisms such as *Staphylococcus aureus*, *Klebsiella pneumoniae*, *Bacillus subtilis*, and *Escherichia coli*. This is not unexpected, as some studies on probiotic LAB have reported the production of bioactive substances by these organisms. These substances, which could be bacteriocins or other antibacterial compounds, could be responsible for the activity shown by the organisms against some test organisms or potentially pathogenic strains. In a study conducted by Sharafi et al. [58], it was reported that *Lactobacillus plantarum* from their study showed antibacterial activity against *E. coli* and *Salmonella* sp., while another strain of the same organism reported by Francois et al. [59], showed good activity against *Klebsiella pneumoniae*, *Pseudomonas* species and *Enterococcus faecalis*. This is also in congruence with a recent study, where potential probiotics (*Enterococcus faecium* ZJUIDS-R1 and *Ligilactobacillus animalis*) exhibited antimicrobial activity against some pathogens, which included *Escherichia coli*,* Salmonella typhimurium*, and *Staphylococcus aureus* [46, 47]. Chaichana et al. [50] and Jiang et al. [54], demonstrated the presence of these genes in the genomes of bacteria isolated from various sources. The biosynthesis of lipids, natural substances, and a variety of secondary metabolites, ranging from signalling molecules to bioactive natural products, is among the important biological roles of T3PKS in bacteria [53]. The in vitro screening and genomic screening confirmed the efficacy of the three LAB from this study as probiotics, with the ability to secrete antimicrobial compounds.

The tolerance of our three potential probiotic organisms to acidic pH 2 is similar to other results, where lactic acid bacteria with probiotic properties such as *Lactobacillus plantarum*, found in Italian foods of animal origin, have been able to withstand acidic pH [60]. Additionally, others in their preliminary work revealed that both *Enterococcus faecium* and *Ligilactobacillus animalis* were viable at pH 1.5 [43, 61]. This metabolic diversity in *Enterococcus* species from rabbit gut is a key characteristic that enables these isolates to survive under stress and physiological conditions [7, 11]. Additionally, all three isolates demonstrated the ability to withstand simulated duodenal juice, which mimics the conditions of the duodenum. This tolerance is a key prerequisite for classifying microorganisms as probiotics. Although viable cell counts declined with prolonged incubation, the isolates remained recoverable, indicating their capacity to survive under such conditions. Similar observations have been reported in previous studies [17].

One of the criteria to determine the potential ability of LAB to attach to the gut region is the assay for cell surface hydrophobicity. This feature suggests the ability of the cell to attach to the lining of the GIT, hence a potentially probiotic organism. All three selected LAB isolates in this study showed a high level of adhesion to hydrocarbons (xylene and n-hexane), with the maximum adhesion recorded for *Enterococcus lactis* UIADO37 (51.5%) followed by *Enterococcus hirae* UIADO30 (50%). The lowest level of adhesion was to *Enterococcus lactis* UIADO22 with a percentage adhesion of 41.8%. The observation from this study agrees with the findings of Collado et al. [62] and Duary et al. [63] who reported a percentage adhesion to hydrocarbon of 44.2% and 25.06%. However, the values obtained in their study are lower than the values obtained in this study.

Comparing the in vitro screening for probiotics using acidic pH, their ability to survive in simulated duodenum juice, and cell surface hydrophobicity with the information from the whole genome sequence analysis, there is a trend of homology in the observation. The carriage of genes such as *dltA*, *dltB*, *dltC*, *dltD*, *dnaK*, *spxA*, *rex*, *perR*, and *glsA* by the LAB reflects their intrinsic capacity for environmental adaptation, stress tolerance, and host interaction traits essential for probiotic functionality further confirming the results of the in vitro screening. The chaperone gene (*dnaK*), part of the HSP70 family, plays a vital role in protein refolding and stabilization during heat and acid stress conditions typically encountered in the gastrointestinal tract. It has been linked to enhanced survival and bile resistance in various lactic acid bacteria, including *Enterococcus* and *Lactobacillus* spp. Likewise, *spxA*, *rex*, and *perR* encode regulatory proteins involved in managing oxidative stress and redox balance, which are crucial for probiotic survival during gut transit and immune system interactions [43] While these genes may also be present in pathogenic bacteria, their presence in probiotic strains is primarily linked to non-pathogenic stress resilience and competitive colonization, rather than virulence [49].

The analysis of the genomes of the three probiotic LAB, revealed the presence of several genes involved in DNA and protein protection and repair, including *uvrA*, *uvrB*, *mutS*, *mutL*, *recN*, *ssb*, *clpP*, *clpX*, *groEL*, *dnaK*. These genes are not indicative of virulence; rather, they reflect mechanisms essential for probiotic survival in the hostile gastrointestinal environment. They correct DNA lesions caused by oxidative stress, bile salts, and acidic pH, which helps to maintain the stability of the genomes [15]. Chaperone-encoding genes such as *clpP*, *clpX*, *groEL* (*HSP60/CPN60*), and *dnaK* (*HSP70*) are highly conserved in lactic acid bacteria and facilitate proper protein folding, degradation of damaged proteins, and response to heat, acid, and osmotic stress, which enable the probiotics to be able to survive in the gastrointestinal tract [7]. Furthermore, *agrB *and* fsrA* were detected in the genomes of the three organisms, while *agrD* was found only in *Enterococcus lactis* UIADO22. These genes encode cell density-dependent regulatory systems that modulate gene expression during environmental adaptation [50]. The *fbp*, a gene which sometimes in conjunction with *mub* genes is responsible for adhesion in *Lactobacillus acidophilus*, was found in the genomes of *Enterococcus lactis* UIADO22 and *Enterococcus lactis* UIADO37 but absent in *Enterococcus hirae* UIADO30.

This study expands current knowledge on probiotics by isolating lactic acid bacteria from the rabbit gastrointestinal tract, underscoring the significance of this source. The three strains identified, *Enterococcus lactis* UIADO22, *Enterococcus lactis* UIADO37, and *Enterococcus hirae* UIADO30, exhibited promising probiotic traits using in vitro and whole genome sequence analysis. These findings highlight their potential for application as safe and functional probiotics, and the need for further evaluation through in vivo studies.

## Conclusion

In this study, Lactic Acid Bacteria isolated from the gastrointestinal tract of domestic rabbits were identified as *Enterococcus lactis* UIADO22, *Enterococcus lactis* UIADO37, and *Enterococcus hirae* UIADO30, and whole genome sequence analysis revealed genes encoding production of bacteriocins, antimicrobial secondary metabolites, and other genes associated with probiotic potential. The genomic data however, reflect only genetic potential and not in vivo expression or functionality. Future studies should integrate transcriptomic, proteomic, and/or metabolomics approaches to bridge the gap between predicted probiotic traits and actual gut activity. 

## Future outlooks and recommendations

Based on these findings, the probiotic LAB isolated from rabbits are considered safe and suitable for use as feed additives in animal production. Consequently, future studies should focus on the quantification and purification of their bacteriocins and antimicrobial compounds to determine effective dosages for probiotic application in animals.

## Supplementary Information

Supplementary Material 1.

## Data Availability

The datasets (whole genome sequences) generated and/or analysed during the current study are available in the NCBI GenBank under the following Accession Numbers SRR34113111, SRR34113110 and SRR34113109.
