# Correction: Functional and genomic insights into probiotic lactic acid bacteria isolated from the gastrointestinal microbiota of domestic rabbits (*Oryctolagus cuniculus*)

**DOI:** 10.1186/s12866-026-05317-9

**Published:** 2026-07-09

**Authors:** Oluwatosin Olubunmi Oladipo, Abimbola Olumide Adekanmbi, Opeyemi U. Lawal, Valeria R. Parreira, Bolaji Fatai Oyeyemi, Olatunji Abubakar Jimoh, Aderemi Akinyemi, Charles Ayorinde Ologunde, Ayonposi Bukola Olaoye, Olugbenga David Oloruntola, Mitra Soni, Harmanpreet Kaur, Lawrence Goodridge

**Affiliations:** 1 Environmental Microbiology and Biotechnology LaboratoryDepartment of Microbiology, University of Ibadan, Ibadan, Nigeria; 2 Molecular Biology and Biotechnology LaboratoryDepartment of Microbiology, University of Ibadan, Ibadan, Nigeria; 3 The Federal Polytechnic Ado-Ekiti, Department of Science TechnologyAdo- Ekiti, Ekiti State Nigeria; 4 University of Windsor, School of the EnvironmentOntario, Canada; 5 Great Lakes Institutes for Environmental Research, University of Windsor, Ontario, Canada; 6 Canadian Research Institute for Food Safety, University of Guelph, Guelph, ON Canada; 7 Agricultural Technology Department, The Federal Polytechnic Ado Ekiti, Ado Ekiti, Nigeria; 8 Cell Biology and Genetics UnitDepartment of Zoology, University of Ilorin, Ilorin, Nigeria; 9 Department of Animal Science, Adekunle Ajasin University, Akungba Akoko, Nigeria

**Correction: BMC Microbiol 26**,** 478 (2026)**

**https://doi.org/10.1186/s12866-026-04871-6**

Following publication of the original article [1], the author reported that the following requests were not addressed in the final version.

Figure 5 Isolate names should be corrected.

From


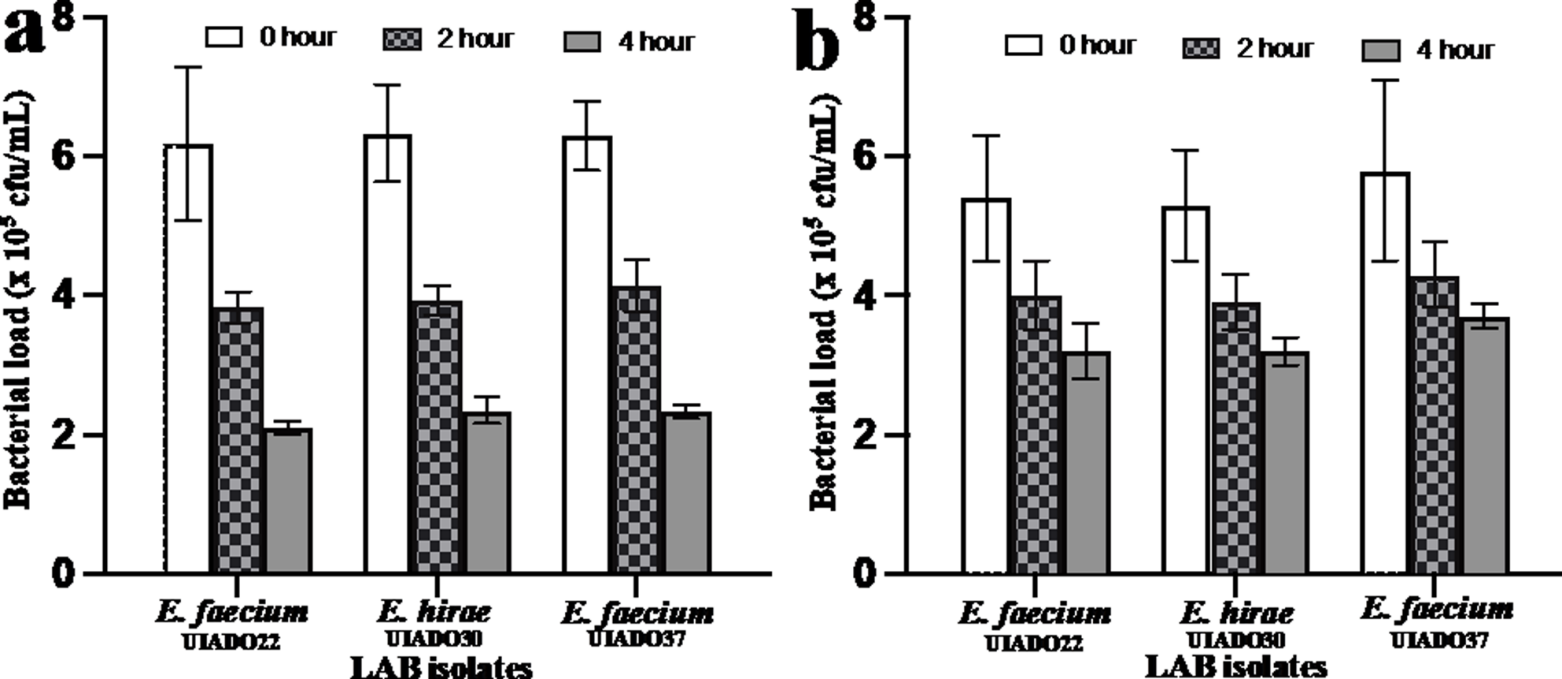


To


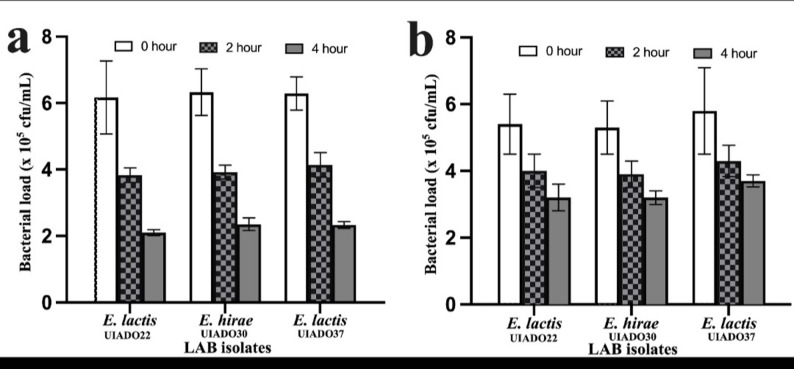


From

**Table 4:** Cell surface hydrophobicity of the LAB isolates

Isolate name	*n*-Hexane	Xylene	% Adhesion
*Enterococcus faecium* UIADO22	40.9	42.6	41.8
*Enterococcus hirae* UIADO30	48.3	51.7	50.0
*Enterococcus durans* UIADO37	52.4	50.5	51.5

To

**Table 4:** Cell surface hydrophobicity of the LAB isolates

Isolate name	n-Hexane	Xylene	% Adhesion
*Enterococcus lactis* UIADO22	40.9	42.6	41.8
*Enterococcus hirae* UIADO30	48.3	51.7	50.0
*Enterococcus lactis* UIADO37	52.4	50.5	51.5

Page 7: ‘’Staphylococcus aureus’’ should be removed.

The original article has been updated.
