# Supplementary material for: Functional and Genomic insights into probiotic Lactic Acid Bacteria isolated from the Gastrointestinal microbiota of domestic rabbits (Oryctolagus cuniculus)
Source: BMC Microbiol. 2026 Apr 9;26:478. doi: 10.1186/s12866-026-04871-6 (PMC13191895; doi:10.1186/s12866-026-04871-6)
Supplement: Supplementary file 1 — Supplementary Material 1. [file 12866_2026_4871_MOESM1_ESM.doc]

Table S1: Antibiotic Susceptibility testing of the LAB isolated from the gut of the rabbits

| Isolate | pen | amp | imi | gen | van | ery | cli |
| --- | --- | --- | --- | --- | --- | --- | --- |
| *Enterococcus faecium* UIADO22 | S | S | S | S | S | S | S |
| *Enterococcus hirae*  UIADO30 | S | S | S | S | S | S | S |
| *Enterococcus durans*  UIADO37 | S | S | S | S | S | S | S |

**KEY: pen: penicillin (10 μg); amp: ampicillin (10 μg); imi: imipenem (10 μg); gen: gentamicin (10 μg); van: vancomycin (30 μg); ery: erythromycin (15 μg); cli: clindamycin (2 μg); S: sensitive**

Table S2: Haemolytic and DNAse activity of the three selected LAB isolates

| Isolate code | Haemolyitc activity | DNAse activity | Phenol tolerance |
| --- | --- | --- | --- |
| *Enterococcus faecium* UIADO22 | Non-haemolytic | No activity | + |
| *Enterococcus hirae*  UIADO30 | Non-haemolytic | No activity | + |
| *Enterococcus durans* UIADO37 | Non-haemolytic | No activity | + |

**KEY: +: Positive (Could tolerate)**

**Table S3: Plasmids in the two genomes of *Enterococcus lactis* UIADO 22 and *Enterococcus lactis*** UIADO 37

| **Genome** | **Plasmid** | **Identity (%)** | **Position in contig (bp)** | **Plasmid type** | **Accession number** | **Known-Match LAB plasmid** |
| --- | --- | --- | --- | --- | --- | --- |
| *Enterococcus lactis* UIADO22 | repUS15 | 99.71 | 27802-28842 | *rep A* (pNB2354p1) | CP004064 | *Enterococcus faecium* |
|  | rep1 | 97.04 | 8225-9641 | *repE* (pAMbeta) | AF007787 | *Enterococcus faecalis* |
| *Enterococcus lactis* UIADO37 | rep1 | 97.04 | 8225-9641 | *repE* (pAMbeta) | AF007787 | *Enterococcus faecalis* |
|  | repUS15 | 99.71 | 39224-40264 | *repA* (pNB2354p1) | CP004064 | *Enterococcus faecium* |

Table S4: Prophage regions in the genomes of the three Probiotic LAB

| **Genome** | **Region** | **Region Position** | **Size(kb)** | **Score** | **Completeness** | **Total protein** | **Closest phage match** |
| --- | --- | --- | --- | --- | --- | --- | --- |
| *Enterococcus lactis* UIADO22 | 1 | 149315-187047 | 37.7 | 150 | intact | 48 | PHAGE_Lister_2389_NC_003291(14) |
| *Enterococcus hirae* UIADO30 | 1 | 613-17702 | 17.0 | 110 | intact | 20 | PHAGE_Entero_vB_IME197_NC_028671(7) |
|  | 2 | 401170-414779 | 13.6 | 30 | incomplete | 25 | PHAGE_Entero_phiFL3A_NC_013648(5) |
|  | 3 | 119664-179253 | 59.5 | 150 | intact | 65 | PHAGE_Entero_vB_IME197_NC_028671(12) |
|  | 4 | 11-15065 | 15 | 100 | intact | 18 | PHAGE_Strept_9874_NC_031023(2) |
| *Enterococcus lactis* UIADO37 | 1 | 149315-187047 | 37.7 | 150 |  | 48 | PHAGE_Lister_2389_NC_003291(14) |
|  |  |  |  |  |  |  |  |
